# Using HIV Surveillance Registry Data to Re-Link Persons to Care: The RSVP Project in San Francisco

**DOI:** 10.1371/journal.pone.0118923

**Published:** 2015-03-06

**Authors:** Kate Buchacz, Miao-Jung Chen, Maree Kay Parisi, Maya Yoshida-Cervantes, Erin Antunez, Viva Delgado, Nicholas J. Moss, Susan Scheer

**Affiliations:** 1 Division of HIV/AIDS Prevention, Centers for Disease Control and Prevention, Atlanta, Georgia, United States of America; 2 San Francisco Department of Public Health, San Francisco, California, United States of America; 3 Alameda County Public Health Department, Oakland, California, United States of America; Rollins School of Public Health, Emory University, UNITED STATES

## Abstract

**Background:**

Persons with unsuppressed HIV viral load (VL) who disengage from care may experience poor clinical outcomes and potentially transmit HIV. We assessed the feasibility and yield of using the San Francisco Department of Public Health (SFDPH) enhanced HIV surveillance system (eHARS) to identify and re-engage such persons in care.

**Methods:**

Using SFDPH eHARS data as of 4/20/2012 (index date), we selected HIV-infected adults who were alive, had no reported VL or CD4 cell count results in the past nine months (proxy for “out-of-care”) and a VL >200 copies/mL drawn nine to 15 months earlier. We prioritized cases residing locally for investigation, and used information from eHARS and medical and public health databases to contact them for interview and referral to the SFDPH linkage services (LINCS). Twelve months later, we matched-back to eHARS data to assess how HIV laboratory reporting delays affected original eligibility, and if persons had any HIV laboratory results performed and reported within 12 months after index date (‘new labs’).

**Results:**

Among 434 eligible persons, 282 were prioritized for investigation, of whom 75 (27%) were interviewed, 79 (28%) could not be located, and 48 (17%) were located out of the area. Among the interviewed, 54 (72%) persons accepted referral to LINCS. Upon match-back to eHARS data, 324 (75%) in total were confirmed as eligible, including 221 (78%) of the investigated; most had new labs.

**Conclusions:**

Among the investigated persons presumed out-of-care, we interviewed and offered LINCS referral to about one-quarter, demonstrating the feasibility but limited yield of our project. Matching to updated surveillance data revealed that a substantial minority did not disengage from care and that most re-engaged in HIV care. Verifying persons’ HIV care status with medical providers and improving timeliness of transfer and cross-jurisdictional sharing of HIV laboratory data may aid future efforts.

## Introduction

The benefits of combination antiretroviral therapy (ART), both for preserving the health of infected persons and for preventing further HIV transmission, have been well established [1–3] and support high-impact combination prevention strategies [4–7] to reduce the burden of HIV/AIDS in the United States (US). As such, the Department of Health and Human Services released revised recommendations in 2012 that all HIV-infected persons initiate ART regardless of immunologic status [8]. Realizing individual- and population-level benefits of ART depends on timely diagnosis of HIV infection followed by linkage to and continuous engagement in care.

National analyses of the continuum of HIV care indicate that substantial proportions of HIV-infected persons are either unaware of their infection or, if aware, fail to stay engaged in care. Nationally, only about 40% of persons living with HIV are consistently engaged in HIV care [9–12], and only 19% to 25% are virally suppressed [9,13]. The percentages of virally suppressed persons in the State of California and among persons living with HIV in San Francisco are estimated to be higher [14,15]. Engagement in continuous HIV care facilitates achieving a suppressed viral load; however, a recent study demonstrated that linkage to a single care visit was not associated with viral suppression [16]. Persons disengaged from HIV care may have unsuppressed viral loads, and could transmit HIV to their sexual and needle-sharing partners [5,17,18]. Therefore, ensuring consistent engagement in HIV care is an important component of comprehensive HIV prevention and disease control activities [19].

The Centers for Disease Control and Prevention (CDC) and local health departments have been using routinely collected HIV surveillance data to monitor progress toward achieving the goals of the National HIV/AIDS Strategy (NHAS) as well other indicators along the continuum of care [11,16,20–23]. For example, in San Francisco, an estimated 50% of newly HIV diagnosed persons achieve viral suppression within 12 months of diagnosis [22], and 80% of all HIV-infected persons are receiving consistent HIV care defined by biannual visits reported to the HIV surveillance program [24].

In addition to monitoring NHAS progress, there is a growing interest in using existing HIV surveillance and other healthcare databases for public health practice and interventions [25,26]. Increasingly, local health departments have been using surveillance data to inform program activities to help link or re-link persons to HIV care [16,26–31]. We report here the findings from our pilot project, the Re-engaging Surveillance-identified Viremic Persons (RSVP) project, which used surveillance data to identify, contact, and re-engage in care HIV-infected persons with unsuppressed HIV viral load who appeared to have disengaged from care in San Francisco.

## Methods

### Overview of the RSVP project

The RSVP project was launched on April 20, 2012 and implemented over the course of a 12-month period by the San Francisco Department of Public Health (SFDPH). Our goals were to: (a) assess the feasibility of using the enhanced HIV/AIDS reporting system (eHARS) to identify persons with unsuppressed plasma HIV RNA viral load (VL) who appeared to have fallen out of HIV care, as evidenced by a gap in HIV VL and CD4 cell count laboratory reports in eHARS; (b) assess the feasibility and utility of using information available in eHARS and additional databases (described below) to contact persons for re-linkage to HIV care; and (c) determine the degree to which contacted persons would engage the services of the SFDPH-sponsored Linkage Integration Comprehensive Services (LINCS) Program for re-linkage to HIV care or assistance with referrals to ancillary services [32]. Eligible persons who were located and contacted by SFDPH staff and agreed to participate in this project met with SFDPH staff at the health department or another convenient location, provided a written informed consent, and completed a 30-minute interviewer-administered baseline survey. Participants received $40 for their time, were offered services of the LINCS, and were invited to participate in a follow-up interview three months later.

### HIV surveillance-related information

California State law requires laboratories to report results from all confirmed HIV-positive antibody tests, CD4 cell counts, and HIV VLs to local health departments with the patient’s name, date of birth, gender, and name and address of the ordering medical provider [33]. Local laboratory reports are entered by the SFDPH staff into eHARS, which is centrally maintained for California by the California Department of Health, Office of AIDS (OA) [33]. The SFDPH has access to information for all HIV cases diagnosed, treated, and reported in San Francisco, including access to laboratory tests performed both inside and outside of San Francisco County and entered into the California eHARS [15]. Laboratory data reported within San Francisco County are >90% complete, accurate, and timely per CDC standards [34]. However, these metrics may be lower in some other California counties. Vital status of HIV-infected Californians is verified by monthly matches with local and California vital statistics departments, and yearly with the National Death Index and the Social Security Death Master File. Information on place of residence for HIV-infected persons in San Francisco is collected at initial HIV diagnosis, and is updated every 18–24 months through medical record review, and when available, through incoming laboratory reports. SFDPH surveillance staff have confidential access to electronic medical records of reported HIV cases at the county public hospital, public health clinics, and many private medical facilities.

### Population of interest, eligibility criteria, and project flow

#### Step 1: RSVP project eligibility

**Fig 1:**
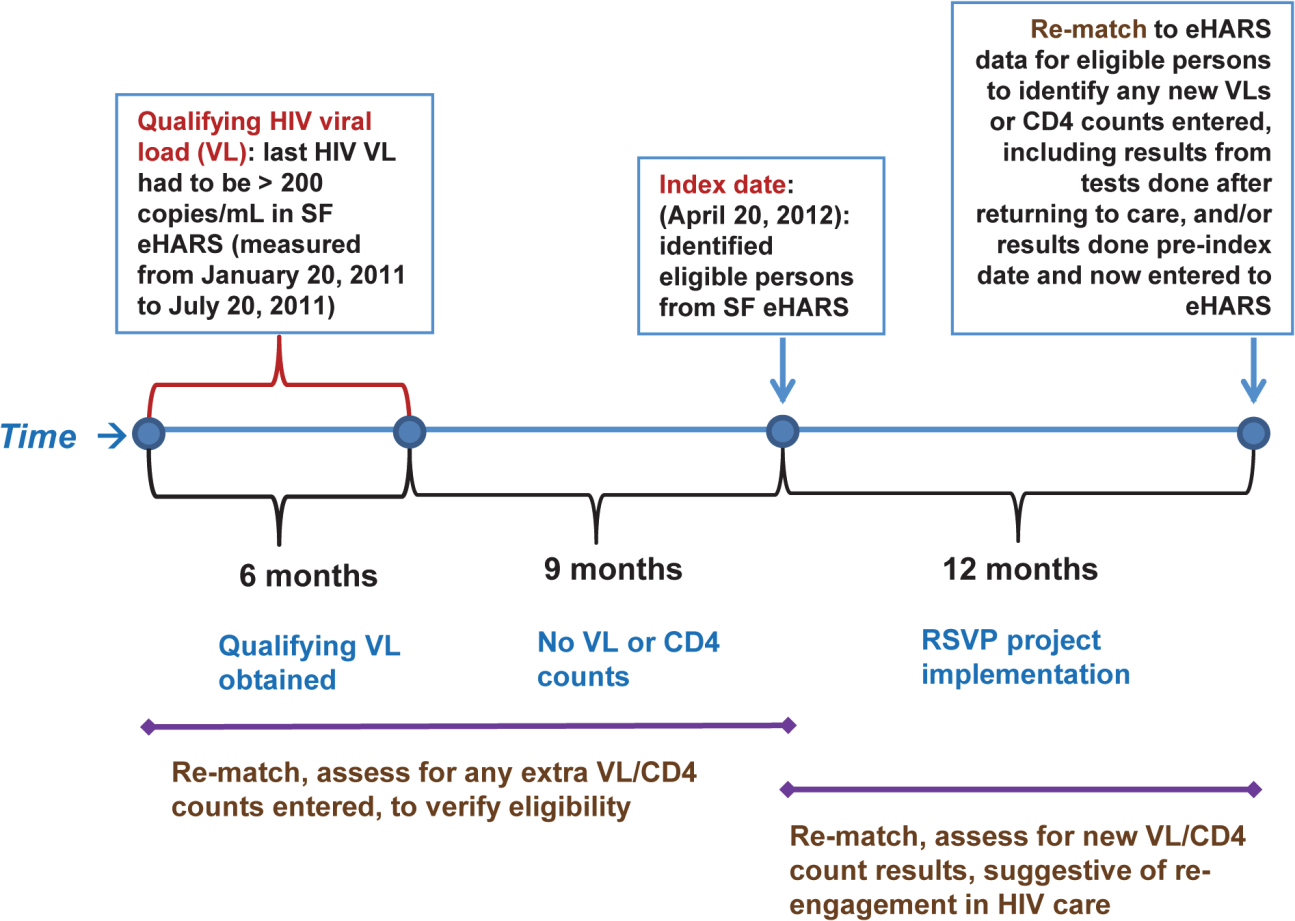
Re-engaging Surveillance-Identified Viremic Persons (RSVP) Project in San Francisco, 2012–2013: Design and Timeline.

We selected as RSVP project-eligible all persons with HIV infection reported to San Francisco eHARS as of April 20, 2012 (“index date”) who met the following inclusion criteria: (a) aged 18 years and older and alive on the index date; (b) had no HIV VL test result or CD4 cell count reported for nine months before the index date; and (c) had an HIV VL > 200 copies/mL at last measurement obtained nine to 15 months earlier (i.e., before the gap in laboratory reports) that is between January 20, 2011 and July 20, 2011 (“qualifying VL”) (Fig. 1). The second and third criteria in combination were used to identify persons who had been in HIV care in the past but appeared to have had a relatively recent but substantial gap in care of at least nine months. These criteria were selected to increase the odds that these viremic persons had actually disengaged from HIV care and that they were still residing in the San Francisco area and could be found. At the time of this project, the Department of Health and Human Services (DHHS) recommended that HIV-infected persons receive HIV laboratory monitoring at least every six months and more frequently (every three months or more often) if viremic or if recently started ART [8,35].

#### Step 2: Selecting persons for public health investigation

We limited our investigation to persons living within San Francisco due to limited SFDPH staffing resources, and because LINCS serviced only persons living locally. To this end, we determined the residence of cases using information on the last known address in eHARS, and the location of the medical provider who drew the qualifying HIV VL. We then investigated and sought to interview those persons who: (a) resided in the Greater San Francisco Bay Area (GBA) defined as San Francisco, Alameda, Contra Costa, San Mateo, Marin, Solano, Napa, Sonoma, Santa Clara, and Santa Cruz counties; (b) had their last HIV VL or CD4 cell count test within the GBA; (c) were able to speak English; and (d) were not incarcerated when contacted for an interview.

#### Step 3. Tracing, contacting, and screening of investigated persons

We reviewed available address information in eHARS (current address, and address at HIV/AIDS diagnosis) for each person selected for public health investigation (also referred to as the “investigated” case). We mailed a letter to the medical provider who ordered the qualifying HIV VL to inform him or her about the RSVP project [36]. Providers could request that their patient not be contacted for any reason, such as relocation away from San Francisco, complicating medical condition, or potential for the project to interfere in the patient-provider relationship.

During the one-year project implementation period, we optimized the methods used to obtain viable contact information. For the majority of investigated cases, we relied on contact information from eHARS or from patients’ electronic medical records maintained by their providers. For persons who required enhanced efforts to be located, we used the SFDPH sexually transmitted disease report database, the California State databases of support services in San Francisco (shelter and substance abuse), a person location service (Lexis/Nexis), and re-reviewed medical records in search of any newly added contact information.

First, we contacted cases by calling each working telephone number up to 10 times; if telephone contact was unsuccessful, a letter was sent inviting the person to contact the SFDPH regarding a “health survey.” A dedicated confidential RSVP project telephone line was established for potentially eligible cases to return calls. Upon telephone contact, we confirmed the person’s identity by self-reported date of birth (or month and year) then scheduled the baseline interview, which explored demographics, HIV care patterns, and reasons for being out of HIV care (survey findings to be presented separately).

### Project evaluation and data analyses

For all eligible cases, we “matched back” records to the San Francisco eHARS data available 12 months after the index date, to determine: (1) if any originally eligible persons were misclassified as “out of care” or viremic based on incomplete lab data at the time of index date; and (2) if any eligible persons re-linked to HIV care within 12 months after the index date as evidenced by new HIV VL or CD4 cell count results in eHARS.

### Data security and confidentiality procedures

All data and analyses for this project were handled in accordance with the security and confidentiality procedures used at the SFDPH for HIV/AIDS surveillance, as required by the State of California Office of AIDS, California state law, and the CDC [36]. Participants were assigned a unique RSVP project identification code used on interview forms, which were kept separate from all personal identifying information. All personal identifying information, including an encrypted electronic file linking a person’s name to RSVP project code, was stored in the secure SFDPH HIV/AIDS surveillance case registry area. No data with personally identifiable information were transferred to the CDC.

### Ethics Statement

The project was determined by the CDC to be a public health practice related to epidemic or endemic disease control activity, rather than human subject research, and was exempt from review by a CDC institutional review board [37]. A HIPAA waiver was not obtained from project participants as HIV surveillance data collected by health departments are HIPAA-exempt [38]. All project participants signed a written informed consent form.

## Results

### Population of interest

Out of 22,395 HIV-infected persons reported in the San Francisco eHARS presumed alive as of April 20, 2012, and residing inside and outside the jurisdiction at the time of their diagnosis, 434 (1.9%) were eligible for the RSVP project (Fig. 1, Table 1). The qualifying HIV VL tests ordered by a city and county public hospital (n = 80, or 18%), private hospital (n = 65, or 15%), private physician (n = 63, or 15%), public health clinic (n = 55, or 13%), Veterans Administration hospital (n = 6, or 1%), and the county jail (n = 5, or 1%). The ordering provider or venue was unknown for 160 (37%) of laboratory reports.

**Table 1:** Characteristics* of the eligible population and investigated cases, the RSVP project, San Francisco, 2012–2013.

	RSVP Project Eligible (N = 434)	Investigated (N = 282)	Investigated, Interviewed (N = 75)	Investigated, Not Interviewed† (N = 207)	p-value^‡^
Age, median years (IQR)	45 (37–51)	44 (36–50)	45 (37–50)	44 (35–51)	0.58
Sex, n (%)					
Male	392 (90)	250 (89)	65 (87)	185 (89)	0.53
Female	42 (10)	32 (11)	10 (13)	22 (11)	
HIV infection risk, n (%)					
Man who has sex with men	366 (84)	228 (81)	64 (85)	164 (79)	0.74
Heterosexual	27 (6)	27 (10)	5 (7)	22 (11)	
Injection drug user	27 (6)	16 (6)	4 (5)	12 (6)	
Other or unknown	14 (3)	11 (4)	2 (3)	9 (4)	
Race/ethnicity, n (%)					
Non-Hispanic white	229 (53)	129 (46)	32 (43)	97 (47)	0.39
Non-Hispanic black	86 (20)	65 (23)	20 (27)	45 (22)	
Non-Hispanic Asian/Pacific Islander	22 (5)	20 (7)	2 (3)	18 (9)	
Hispanic	82 (19)	63 (22)	20 (27)	43 (21)	
Other	13 (3)	4 (1)	1 (1)	3 (1)	
Unknown	2 (0)	1 (0)	0 (0)	1 (0)	
Year of HIV diagnosis, n (%)					
≤ 1994	85 (20)	42 (15)	14 (19)	28 (14)	0.53
1995–2000	89 (21)	47 (17)	11 (15)	36 (17)	
≥ 2001	260 (60)	193 (68)	50 (67)	143 (69)	
County of residence, n (%)					
San Francisco (SF)	260 (60)	211 (75)	59 (79)	152 (73)	0.11
Greater Bay Area other than SF	71 (16)	62 (22)	15 (20)	47 (23)	
Other California county	61 (14)	1 (0)	1 (1)	0 (0)	
Other State	33 (8)	0 (0)	0 (0)	0 (0)	
Missing	9 (2)	8 (3)	0 (0)	8 (4)	
Qualifying^§^ viral load, n (%)					
> 200–999 copies/mL	70 (16)	34 (12)	11 (15)	23 (11)	0.20
1,000–9,999 copies/mL	129 (30)	93 (33)	27 (36)	66 (32)	
10,000–100,000 copies/mL	164 (38)	112 (40)	31 (41)	81 (39)	
> 100,000 copies/mL	71 (16)	43 (15)	6 (8)	37 (18)	
CD4 count ^ǁ^, n (%)					
< 200 cells/mm^3^	112 (26)	70 (25)	13 (17)	57 (28)	0.09
200–349 cells/mm^3^	77 (18)	51 (18)	21 (28)	30 (14)	
350–499 cells/mm^3^	68 (16)	44 (16)	11 (15)	33 (16)	
500+ cells/mm^3^	138 (32)	97 (34)	26 (35)	71 (34)	
Missing	39 (9)	20 (7)	4 (5)	16 (8)	
Any ARV use, n (%)					
Yes	246 (57)	145 (51)	39 (52)	106 (51)	1.00
No	188 (43)	137 (49)	36 (48)	101 (49)	

* Based on eHARS data as of index date, 04/20/2012, unless otherwise specified.

† The 207 persons who were investigated but ultimately not interviewed included 59 (29%) who were found to be either ineligible during investigation (non-English speakers), deceased, or residing outside of the Greater Bay Area.

^‡^ p-values comparing the 75 persons interviewed and 207 not interviewed were calculated usingWilcoxon-Mann-Whitney test or Fisher’s exact test.

§ Qualifying HIV viral load: last measurement from January 20, 2011 to July 20, 2011 time period.

ǁ Closest CD4 cell count result obtained between six months prior and three months after the qualifying viral load collection date.

Abbreviations: ARV; antiviral; IQR; interquartile range.

**Fig 2:**
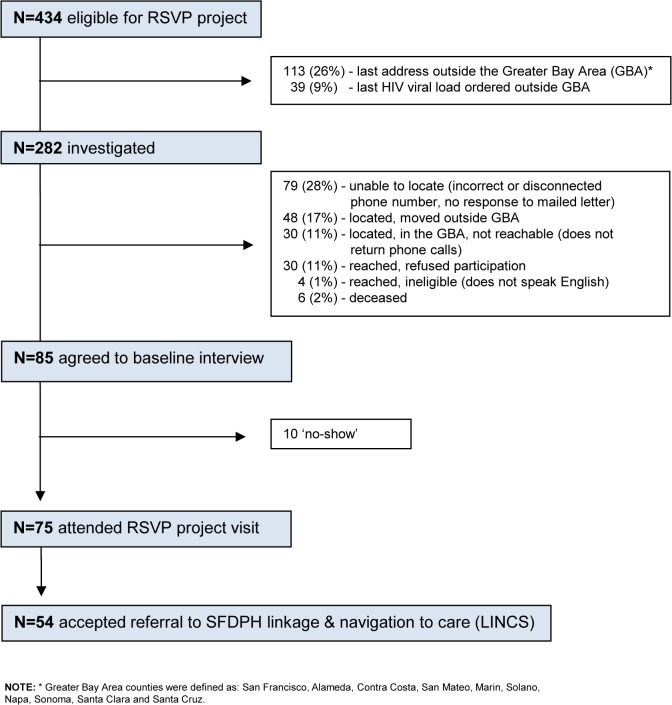
RSVP Project Implementation Flowchart.

Based on review of address and available provider information in eHARS, 152 (35%) of 434 RSVP project-eligible persons were excluded from further investigation: 113 living outside of GBA, and 39 who had their qualifying HIV VL test ordered by a provider outside of GBA (Fig. 2). The remaining 282 cases selected for investigation were similar to the 434 persons eligible for the RSVP project with respect to most socio-demographic and clinical variables (Table 1). A similar distribution of provider types ordering the qualifying HIV VL (data not shown) was also observed, with the exception that fewer (16%) had an unknown source. Only three providers responded to SFDPH’s informational letter and asked to review the list of patients identified for RSVP project from their clinic site; none recommended that SFDPH remove any patients from consideration for RSVP project.

### Results for RSVP project-investigated persons

**Fig 3:**
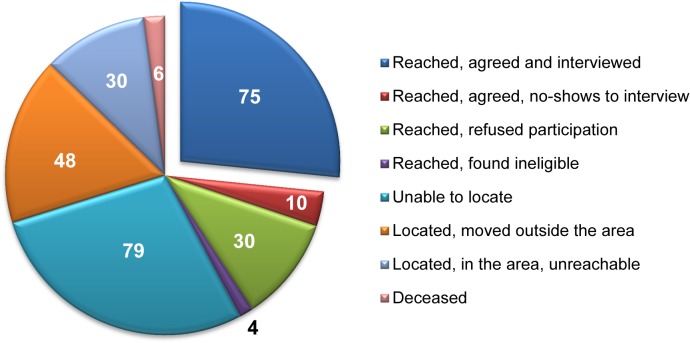
Disposition for 282 Investigated Cases, the RSVP Project, San Francisco, 2012–2013 (n = 282).

Of the 282 persons whom we investigated and attempted to contact, 79 (28%) could not be located, 48 (17%) had moved outside the GBA, and 30 (11%) did not respond to repeated attempts to contact them (Fig. 2). Forty-four individuals (16%) were successfully contacted but not interviewed (30 refused to participate in the project, four were unable to speak English, and 10 agreed to be interviewed but did not subsequently show up to be interviewed). Six persons (2%) were found to be deceased. The remaining 75 individuals (27%) completed the baseline interview. The relative fractions of persons who were investigated and reached, located but not reached, and not located are displayed in Fig. 3.

We sought updated contact information beyond what was available as of the index date in eHARS or in the patient’s electronic medical record maintained by their provider for over half (n = 161) of the 282 patients we investigated; 107 patients required further enhanced database search efforts by SFDPH staff to be contacted or located.

Among the 75 persons interviewed, 87% were male, 85% gay, bisexual or other men who had sex with men (MSM), 43% non-Hispanic white, and 79% had San Francisco as their county of last residence in eHARS. Their characteristics were not statistically significantly different from the 207 persons who were also investigated but could not be interviewed (Table 1).

### eHARS match back: confirmation of eligibility at index date

Upon match back to eHARS 12 months after the index date, 110 (25%) of the 434 persons eligible for RSVP project had additional laboratory results available in eHARS that were not present at the index date. Therefore, these persons were either in HIV care or were not viremic at last VL measurement pre-index date and, in retrospect, were not RSVP project-eligible. Of these 110 persons, 107 had HIV VL or CD4 cell count result(s) performed during the nine months before index date but entered into eHARS in the 12 months after the index date and three persons had last HIV VL < 200 copies/mL. Notably, 61 (22%) of the 282 investigated cases and 25 (33%) of the 75 interviewed persons had laboratory data available after the index date and were, in retrospect, not RSVP project-eligible (Table 2).

**Table 2:** Match-back to eHARS data available 12 months after index date to confirm persons’ original eligibility and to assess re-engagement in care based on new HIV laboratory data, the RSVP Project, San Francisco, 2012–2013.

	RSVP Project-eligible	Investigated	Interviewed
***Eligible persons***	N = 434	N = 282	N = 75
Confirm eligibility at index date			
Not eligible*	110 (25%)	61 (22%)	25 (33%)
***Confirmed eligible***	N = 324	N = 221	N = 50
Assess re-linkage to care within 12 months after index date			
***Among eligible persons***			
≥ 1 VL or CD4 cell count	249 (57%)	181 (64%)	62 (83%)
≥ 2 labs (VL or CD4 cell count) ≥ 90 days apart	104 (24%)	80 (28%)	35 (47%)
***Among confirmed-eligible***			
≥ 1 VL or CD4 cell count	171 (53%)	134 (61%)	41 (82%)
≥ 2 labs (VL or CD4 cell count) ≥ 90 days apart	73 (23%)	60 (27%)	24 (48%)

NOTE: VL, viral load; index date was April 20, 2012.

* Subset of persons originally thought to be eligible for the RSVP project who, in retrospect, were not eligible based on additional laboratory results performed before index date but only reported to eHARS during the 12 months after the index date.

### eHARS match back: re-engagement in HIV care during the 12 months after index date

Among the 434 persons eligible for participation in the RSVP project, 249 (57%) had at least one new HIV VL or CD4 cell count performed and reported to eHARS during 12 months after index date and 104 (24%) had two or more laboratory tests at least 90 days apart during this 12-month period (Table 2). These percentages, suggestive of re-engagement in HIV care, were somewhat higher for the persons who were investigated and interviewed (Table 2). After excluding the 110 persons who, in retrospect, due to delayed reporting of HIV VL and CD4 cell count data did not meet the original eligibility criteria, the percentages with one or more laboratory test result among the remaining 324 confirmed eligible persons were similar to those reported for 434 persons and similar comparing the relevant subsets (i.e., total, investigated, and interviewed).

With respect to the timing of the new HIV laboratory measurements, among 75 persons interviewed, 13 (17%) did not have any laboratory test(s) reported after index date, 54 (72%) had a laboratory test after the index date and before their RSVP project visit, and eight (11%) had a laboratory test only after their RSVP project visit.

### Use of LINCS services by interviewed persons

Of the 75 interviewed persons, 54 (72%) accepted a referral to LINCS. LINCS staff determined that 29 (54%) of these persons had truly fallen out of HIV care and therefore attempted to contact these persons to offer re-engagement in care support. To date, 19 (66%) persons have been successfully contacted by LINCS staff and 15 linked to care, of whom 12 have been documented as having attended a new primary HIV care appointment; three who were living outside of San Francisco and one who became incarcerated after his interview. The remaining 25 persons were determined by LINCS staff to be engaged in HIV care per self-report or from information available in the SFDPH’s clinical record database and were offered referrals to ancillary services (e.g., social services, mental health care).

## Discussion

The RSVP project focused on a high-priority HIV-infected population: persons who were out-of-care and viremic, and thus, at increased risk for poor clinical outcomes and potentially transmitting HIV to others. Nonetheless, having previously received HIV care, these persons were possibly more willing to re-link to treatment services if provided additional support [39,40]. Starting with routinely collected eHARS data in San Francisco, we were able to identify and investigate these persons who appeared to be out of HIV care for at least nine months, generate dispositions for all of them, but ultimately reached and interviewed only a small subset: 75 (27%) of 282 persons investigated. Many persons who we believed resided locally had moved outside the GBA, or could not be located or reached. In retrospect, based on updated laboratory data reported to eHARS, approximately 25% of all persons originally identified as eligible for the RSVP project were actually not eligible (i.e., had evidence of recent HIV care). Despite the project’s modest yield, the majority of interviewed persons accepted referral to the LINCS program, and some benefited from LINCS services with re-engaging to HIV care.

Our approach bore some similarities to HIV linkage and re-engagement programs reported by other health departments [16,27–30], and yielded several lessons that may guide further re-linkage-to-care activities in San Francisco and elsewhere. The RSVP project was resource-intensive in staff time required to obtain current contact information and find eligible persons. The HIV surveillance registry did not contain sufficient information to locate persons; phone numbers were not (and continue not to be) collected, and until recently, the latest address information collected was not associated with a date, and thus, potentially outdated to an unknown extent. This necessitated searches for phone numbers and address information in medical records from the clinical provider who ordered the last HIV VL. When such contact information was unavailable or unproductive, we used additional databases to find locating information, including other disease registries and a commercial person-search database. We also found that a substantial minority of persons we attempted to locate were homeless or transitionally housed, transient, and frequently had non-working phone numbers, making reaching them by phone or conventional postal service challenging. Notably, through our eHARS match-back procedure one year later, we found that over half of persons eligible for RSVP project participation had at least one new HIV VL or CD4 cell count reported to eHARS during the 12-month period after the index date in April 2012, suggesting that these persons had re-engaged in HIV care or were never truly out of care but just had sporadic HIV laboratory measurements performed either within or outside the jurisdiction [12,15,41].

The success rates of our and others’ re-linkage programs likely varied due to: (i) differences in definitions of the eligible population, particularly whether or not local residence was required [27] and duration of time since last documented HIV laboratory measurement [30]; (ii) completeness and timeliness of HIV laboratory and address information in local surveillance registries and other databases [16]; (iii) degree of collaboration and information exchange between the Department of Health and medical providers for identifying and tracing people who may have fallen out of HIV care [42]; and (iv) actual rates of retention in HIV care, geography, mobility, and HIV care-seeking patterns of persons in different localities [20–22,41].

Our project was subject to several limitations. Laboratory results in the San Francisco surveillance registry are reported with some delay for a fraction of persons, particularly if they have received testing in counties outside of San Francisco; these counties transfer results to the California Department of Health for processing before making them available to the SFDPH. Although we allowed for a nine-month period without laboratory measurements as an eligibility criterion in our project, laboratory reporting delays still impaired our ability to accurately identify eligible persons. The yield of our project was also lowered by searching for persons who, rather than being out of HIV care, were instead undergoing HIV laboratory testing either less frequently within the GBA, or outside the GBA. Although we attempted to exclude all persons who appeared to have moved out of the GBA from investigation, not all could be excluded *a priori* due to outdated information in the surveillance registry regarding their residential address or missing information about the HIV provider who drew the qualifying VL. In the course of our investigation, we found that a substantial fraction of persons had moved out of the GBA, as was the case for a similar health department project in the Seattle area [31]. Our investigative and outreach efforts were multipronged and concurrent and we were unable to systematically assess the source of information which led to a successful patient contact, including his/her returning a call to the RSVP project staff.

Furthermore, we did not seek and cannot determine the extent to which re-engagement was attributable to contact with RSVP project staff or support provided by LINCS. All eligible persons, whether interviewed or not, received at least one phone call inviting them to enroll in a Health Department project, which may have affected their care-seeking behavior. Also, persons who were interviewed may have been more motivated to resume HIV care than those who were not interviewed. Individuals who were interviewed and engaged with LINCS may have had greater unmet needs than those who were interviewed but did not engage with LINCS.

The RSVP project was essentially a pilot project, had no dedicated budget, and was conducted with no additional staffing except for a part-time CDC assignee who assisted in its design and launch. We did not systematically track and document how resource intensive this project was. As in other studies and pilot projects of this type [29,30,39,43,44], the RSVP project benefitted from leveraging HIV surveillance and LINCS program staff resources to offer a difficult to reach population services they needed rather than just an interview about reasons for disengaging from HIV care. Nonetheless, we relied on existing SFDPH surveillance and public health investigator staff and our success in locating and interviewing eligible persons was likely less than what it would have been had additional dedicated staffing and funding resources been available.

This pilot project provided important information to the health department about the logistics, utility, and yield of using HIV surveillance data to identify and trace persons who appeared to have disengaged from HIV care. In summary, examining routinely reported HIV laboratory reports in an HIV/AIDS surveillance registry was a feasible, albeit imperfect, method to identify persons presumed lost to HIV care. In selecting eligible persons from the surveillance registry, we had to contend with incomplete or outdated HIV case contact information, and the frequent absence of adequately detailed information regarding medical providers who ordered the last HIV VL test. As health departments continue to develop enhanced electronic laboratory reporting for all HIV-related test results collected in the course of care after HIV diagnosis, the utility of HIV surveillance data to investigate, contact, and re-engage persons in care will continue to improve. Based on our RSVP project experience, we recommend that future projects build upon partnerships of health departments and medical providers, and utilize upfront information from both HIV surveillance and medical records to better focus on persons who, based on available information from all sources, still live in the jurisdiction and are not receiving care elsewhere. Strengthening the framework [26,36] for sharing securely and in a timely fashion surveillance data between counties and states may improve local health departments’ success in routinely using HIV surveillance data to identify and re-engage people in HIV care.
